# Comment on: "Economic Burden of Thalassemia Major in Iran, 2015"

**Published:** 2016-11-14

**Authors:** Satar Rezaei, Behzad Karami Matin, Mohammad Hajizadeh

**Affiliations:** ^a^ Research Center for Environmental Determinants of Health, Kermanshah University of Medical Sciences, Kermanshah, Iran.; ^b^ School of Health Administration, Faculty of Health Professions, Dalhousie University, Halifax, Canada

## Dear Editor-in-Chief

We read with interest the paper by Esmaeilzadeh et al. titled “Economic Burden of Thalassemia Major in Iran, 2015” published in the Journal of Research in Health Sciences ^1^. It provides a convincing answer to an important research question. We would like to highlight some points about the paper that may merit attention.

First, although the authors aimed to estimate the economic burden of thalassemia major from the society perspective, mortality cost associated with the disease was not considered as a 'cost' to society in the study. In fact, based on the current literature ^2-6^, more than 50% of total costs was associated with mortality costs. For example, a study by Daroudi et al.^3^ suggested that mortality costs accounted for 77% of total costs of breast cancer in Iran in 2010. We believe that the study underestimate the costs of thalassemia major in Iran. Therefore, it should have been mentioned as one of the study limitations.

Second, the authors mentioned that they calculated direct, indirect and intangible costs when they calculated total costs of thalassemia major. However, there is no information on intangible costs in the results section. It seems that the authors did not consider intangible costs when they estimated total costs of the diseases even though these costs were one of the main components of total cost of the disease from societal perspective. For example, a study by Rezaei et al.^7^ showed that intangible costs were responsible for 17.27% of total costs of economic burden of road traffic crashes (RTC) in Iran in 2009.

Third, the method section of the paper states that the prevalence approach was used to estimate the economic burden of thalassemia major. Nevertheless, the use of this method in the study is not clear throughout the paper because the authors did not mention which part of the findings obtained from using this approach.

Fourth, the authors estimated total costs associated with thalassemia major for 198 patients in 2015. Hence, the title of article should not refer to economic burden of thalassemia major in Iran as a whole. This could have been mentioned as one of the limitations of the study.

Fifth, similar to previous studies ^3,7,8^, it would be useful to present direct healthcare costs, direct non-healthcare costs, patient time costs and costs of premature death in separate table.

Satar Rezaei (PhD)^a^, Behzad Karami Matin (PhD)^a^, Mohammad Hajizadeh (PhD)^b^*

^a^ *Research Center for Environmental Determinants of Health, Kermanshah University of Medical Sciences, Kermanshah, Iran.*

^b^ School of Health Administration, Faculty of Health Professions, Dalhousie University, Halifax, Canada

***Correspondence to:*** *Mohammad Hajizadeh (PhD)*

***E-mail:*** m.hajizadeh@dal.ca

## Reply

Thanks for writing the article "Economic Burden of Thalassemia Major in Iran, 2015". Hereby, the authors respond to the problems stated in Comment on: "Economic Burden of Thalassemia Major in Iran, 2015"

In this study, the economic burden of thalassemia for using in planning and policy-making has been estimated both with and without taking into account the costs associated with premature deaths and disabilities. The results related to intangible costs have been presented below Table 2. In addition, the overall results in which the intangible costs as well as the costs of premature death are included can be seen in the second paragraph of the discussion section. Regarding the intangible costs of accidents pointed in this letter, it is important to note that intangible costs or premature death caused by diseases are not comparable in any way. For example, a disease such as rabies that causes the patient to die within a few days does not create much intangible costs. On the contrary, a disease such as cancer with which that patient may live several years might cause substantial intangible costs. Some parts of the article that contains the expressed results:

“According to Naghavi, Years Lost due to Disability associated with thalassemia is 25%. Given the per capita GDP (Gross Domestic Product) of $ 5442 for Iran in 2014, it can be said that, in addition to the costs expressed in Table 2, almost all patients lose $1360.5 a year for costs associated with pain and suffering caused by the disease.

Taking into consideration the life expectancy of 50 years for thalassemic patients 24, the annual cost of the lost opportunity due to premature death in these patients will be equal to $ 2558.1. By adding the lost welfare costs as well as the direct and indirect treatment costs, the annual and the lifetime expenses of a thalassemic patient (without discount fees) were estimated $ 12,240.4 and $612,020.0”

In the method of estimating the costs based on the prevalence -based approach, the annual costs of the disease are estimated^1^, referred to in the methodology section. In the cost estimation method, it has been clearly stated how the annual costs were collected (one strength of this study was to estimate the costs very accurately).

According to the results, 85% of the costs of thalassemia is paid by the insurance companies and the government, much of which relate to drugs and transfusion that the patients receive. Given that the payers are the same in different regions, the payments by the government and insurance companies cannot vary too much. Regarding the costs that the patients pay, the highest share of costs relates to the lost time for blood collection and testing that can be somewhat similar for the patients. On the other hand, similar studies conducted in Thailand and Taiwan with sample sizes smaller than ours, the heading "Economic burden of thalassemia in Thailand and Taiwan" had been used,^2^

Another strength of this study was to present the costs in terms of age groups (Table 1), cost resources (Table 2) and cost payers (Figure 1) that can be really helpful in controlling the costs. On the other hand, it was pointed out in the discussion and conclusion section that how much of the costs related to intangible costs and premature deaths. (Knowing how much of the costs were direct and how much were indirect would not help the policy-makers to reduce the costs. To control the costs, policymakers need to be aware of the costs generated based on the cost sources. On the other hand, readers who are familiar with cost-related issues can easily get informed of the direct and indirect costs according to Table 2).

Firooz Esmaeilzadeh (MSc)^a^, Batoul Ahmadi (PhD)^a^*

^a^ *Department of Health Management & Economics, School of Public Health, Tehran University of Medical Sciences, Tehran, Iran*

***E*** ***-*** ***mail:*** *ahmadiba@sina.tums.ac.ir*
